# Viral and immunological markers of HIV-associated Kaposi sarcoma recurrence

**DOI:** 10.1371/journal.pone.0254177

**Published:** 2021-07-02

**Authors:** Owen Ngalamika, Marie Claire Mukasine, Musonda Kawimbe, Faheema Vally

**Affiliations:** 1 Dermatology and Venereology Division, Adult University Teaching Hospital, Lusaka, Zambia; 2 University of Zambia School of Medicine, Lusaka, Zambia; 3 HHV8 Research Molecular Virology Laboratory, University Teaching Hospital, Lusaka, Zambia; University of North Carolina at Chapel Hill, UNITED STATES

## Abstract

Kaposi sarcoma (KS) is an AIDS-defining angio-proliferative malignancy highly prevalent in Sub-Saharan Africa. The main objective of this study was to determine the factors associated with recurrence of HIV-associated KS. We recruited a cohort of individuals on antiretroviral therapy who were in remission for HIV-associated KS after undergoing cytotoxic cancer chemotherapy. Collected variables included sociodemographic and clinical parameters, cytokines and chemokines, HIV viral loads, and CD4 counts. Compared to individuals who had KS recurrence, IL-5 was significantly higher at time of follow-up in individuals who had sustained remission (22.7pg/ml *vs*. 2.4pg/ml; p = 0.02); IL-6 was significantly higher at baseline and time of follow-up in individuals who had sustained remission, (18.4pg/ml *vs*. 0pg/ml; p = 0.01) and (18.0pg/ml *vs*. 0.18pg/ml; p = 0.03) respectively; IP-10 was significantly lower at baseline and at time of follow-up in individuals who had sustained remission, (534pg/ml *vs*. 920pg/ml; p = 0.04) and (446pg/ml *vs*.1098pg/ml; p = 0.01) respectively; while HIV viral load was significantly lower at baseline and at time of follow-up in individuals who had sustained remission, (0copies/ml *vs*. 113copies/ml; p = 0.004) and (0copies/ml *vs*. 152copies/ml; p = 0.025) respectively. Plasma levels of IL-5, IL-6, and IP-10 are associated with recurrence of HIV-associated KS, while persistently detectable HIV viral loads increase the risk of KS recurrence.

## Introduction

The vascular malignancy Kaposi sarcoma (KS) is highly prevalent in sub-Saharan Africa (SSA) [1]. It is caused by the Kaposi sarcoma-associated herpes virus (KSHV) which is also highly prevalent in many SSA countries [2]. HIV infection is a risk factor for KS development in individuals who are infected with KSHV [3]. In fact, KS is considered an AIDS-defining malignancy in HIV-infected individuals [4].

The two prevalent forms of KS in SSA include endemic KS which predominantly affects HIV-uninfected males of SSA, and epidemic or HIV/AIDS-associated KS which is a result of HIV-induced immunosuppression [5]. HIV-associated KS is by far the most common form of KS and is treated with antiretroviral therapy (ART) with or without cytotoxic cancer chemotherapy [6]. Commonly used chemotherapy regimens in many SSA countries including Zambia are Adriamycin/Doxorubicin plus Bleomycin plus Vincristine (ABV) as first-line, while Paclitaxel is commonly used for individuals who do not respond to first-line treatment or those who have KS recurrence after an initial response to first-line treatment. Liposomal anthracyclines such as liposomal doxorubicin are a preferred first-line treatment when available.

Clinical outcomes to treatment of HIV-associated KS are variable, with poor treatment response and high disease recurrence rates observed previously. In a previous study, we found that only about 47 percent of HIV-associated KS patients undergoing first-line chemotherapy (ABV) had a complete response to treatment, while 53 percent had a partial response [7]. In addition, almost half of the individuals who initially had a complete response subsequently had KS recurrence within 3 months of completing treatment.

Factors associated with KS disease re-development after initial successful treatment are poorly understood. However, several clinical, immunological, and virological factors have been associated with KS development and treatment outcomes. Presence of nodular lesions as opposed to patches and plaques have been observed to be associated with advanced disease and poor treatment response in KS patients [6]. High plasma levels of interleukin 6 (IL-6) have been observed to be associated with increased KSHV viral replication as well as the clinical manifestations of HIV-associated KS [8]. Interferons (IFN) including IFNγ are known to play an important role in the control of viral infections including KSHV [9]. Low CD4 counts and high HIV viral loads are known risk factors for development and progression of HIV-associated KS [10].

The aim of this study was to determine factors that are associated with re-development of HIV-associated KS in individuals who were on ART and in remission for KS after previous successful treatment with chemotherapy. The factors that were investigated included clinical, sociodemographic, immunological (cytokines and chemokines), and HIV-related.

## Materials and methods

### Study population and samples

This was a prospective study that recruited a cohort of individuals who had been previously successfully treated and were in remission for HIV-associated KS. These individuals were all HIV positive and on ART for at least 6 months at baseline. All these individuals were treated in an outpatient setting and had no history of visceral involvement. They had all been previously treated with the commonly available first-line chemotherapy regimen (Doxorubicin, Bleomycin, and Vincristine). At the time of recruitment, these individuals had completed at least 6 cycles of chemotherapy (given 3 weeks apart) and were in remission for KS, with no evidence of disease. They were followed up until they experienced KS recurrence, or for a maximum period of 12 months. The main objective of the study was to determine if and how cytokines/chemokines and/or HIV-related parameters are associated with recurrence of chemotherapy-treated HIV-associated KS.

At baseline, sociodemographic and clinical information were collected. The patients were then followed up monthly in the first 4 months then at 6 months, then 9 months and then at 12 months to observe for any signs of disease recurrence. Patients also came in at unscheduled follow-up time points. The study follow-up was ended and second-line chemotherapy initiated for individuals that experienced KS recurrence during the follow-up period. Venous whole blood was collected in EDTA vacutainer bottles at baseline and at follow-up visits. Plasma was separated from the whole blood and stored at -80°C until analysis. For this study, analyzed samples included those collected at baseline and at time of KS recurrence for individuals who experienced KS re-development, and at 3 months of follow-up (post-remission) or the latest follow-up (up to 12 months) for those who had sustained remission. This study was approved by the University of Zambia Biomedical Research Ethics Committee (Ref. No. 019-07-18) and the Zambian National Health Research Authority. Written informed consent was obtained from each study participant before enrollment into the study.

### HIV viral load testing

We performed HIV viral load at baseline and at the follow-up time point. The HIV-1 plasma viral load was measured using Aptima HIV-1 Quant Dx Assay kit on the Hologic Panther (Hologic) according to the manufacturer’s protocol.

### CD4 T cell counts

CD4 counts were performed at baseline and at the follow-up time point. The BD TriTest kit (BD biosciences) was used according to the manufacturer’s protocol, and counts were determined on a BD FACSCalibur (BD Biosciences).

### Cytokine and chemokine quantification

Plasma cytokines and chemokines were quantified using the MILLIPLEX MAP human cytokine/chemokine magnetic bead panel kits according to the manufacturer’s instructions (EMD Millipore). All assays were performed in duplicate, with the mean value obtained for final analysis. The following cytokines and chemokines were quantified: Interferon gamma (IFNγ), Interleukin 4 (IL-4), Interleukin 5 (IL-5), Interleukin 6 (IL-6), Interferon-inducible protein 10 (IP-10), and Vascular endothelial growth factor (VEGF). Data was quantified on a Luminex MAGPIX instrument (Luminex Corporation) with xPONENT version 4.3 software.

### Statistical analysis

We used descriptive statistics to analyze baseline characteristics. Continuous variables are presented as median and interquartile range, while categorical or binary variables are presented as percentages.

We used univariate cox regression to determine the association between clinical and sociodemographic predictors with time to KS recurrence. None of the variables satisfied the requirements (p value <0.1) to be included in the multivariate model.

The Wilcoxon Rank-sum test was used to compare differences in cytokines, chemokines, CD4 counts, and HIV viral loads between groups at baseline and at time of follow-up. The Wilcoxon matched-pairs signed-rank test was used to compare paired data within groups. The Spearman’s rank correlation was used to determine existence of a correlation between CD4 counts and HIV viral loads.

## Results

### Characteristics of study participants

We analyzed a total number of 78 samples from a cohort of 39 participants for cytokines, chemokines, CD4 counts, and HIV viral loads. Twenty-seven (27) of these participants were in remission for KS while 12 of them had KS recurrence during the follow-up period (Table 1). We also determined the potential clinical and sociodemographic predictors of KS recurrence for these 39 participants.

**Table 1:** Baseline characteristics of study participants by outcome.

	No KS Recurrence (N = 27)	KS Recurrence (N = 12)
Age (Years)	40 [34–44]	39 [33–42]
Males	19 (70.4%)	8 (66.7%)
Smoking	5 (18.5%)	0 (0%)
Alcohol	6 (22.2%)	2 (16.7%)
Chemotherapy cycle at which improvement noted	3 [2–4]	3 [3–3.5]
Duration of HIV infection (Months)	36 [12–96]	21 [14–42]
Duration of ART uptake (Months)	24 [7–60]	18 [12–31.5]
Lymphedema present	7 (26.9%)	1 (10%)
Mucous membranes were involved	1 (0.04%)	0 (0%)

### Clinical and sociodemographic predictors of KS recurrence

Of all the clinical and sociodemographic characteristics collected and analyzed, there was none that was significantly associated with KS recurrence (Table 2).

**Table 2:** Univariate cox regression on clinical and sociodemographic predictors of KS recurrence.

	Hazard Ratio	95% Confidence Interval	p value
Age	0.99	0.91–1.06	0.74
Male	0.79	0.24–2.62	0.70
Alcohol	0.67	0.15–3.09	0.61
Smoking	6.4*10^-17	0–0	1.00
History of Multiple KS lesions	0.57	0.12–2.61	0.47
History of Painful Lesions	0.51	0.15–1.74	0.28
History of Bleeding Lesions	1.78	0.53–6.00	0.35
Number of Chemotherapy Cycles	1.35	0.74–2.46	0.32
Months Since HIV Diagnosis	0.99	0.97–1.01	0.26
Months on ART	0.99	0.97–1.01	0.33
Presence of Lymphedema	0.30	0.04–2.39	0.26
History of Mucous Membrane Involvement	4.4*10^-15	0–0	1.00

### Comparison of cytokines and chemokines

**Fig 1:**
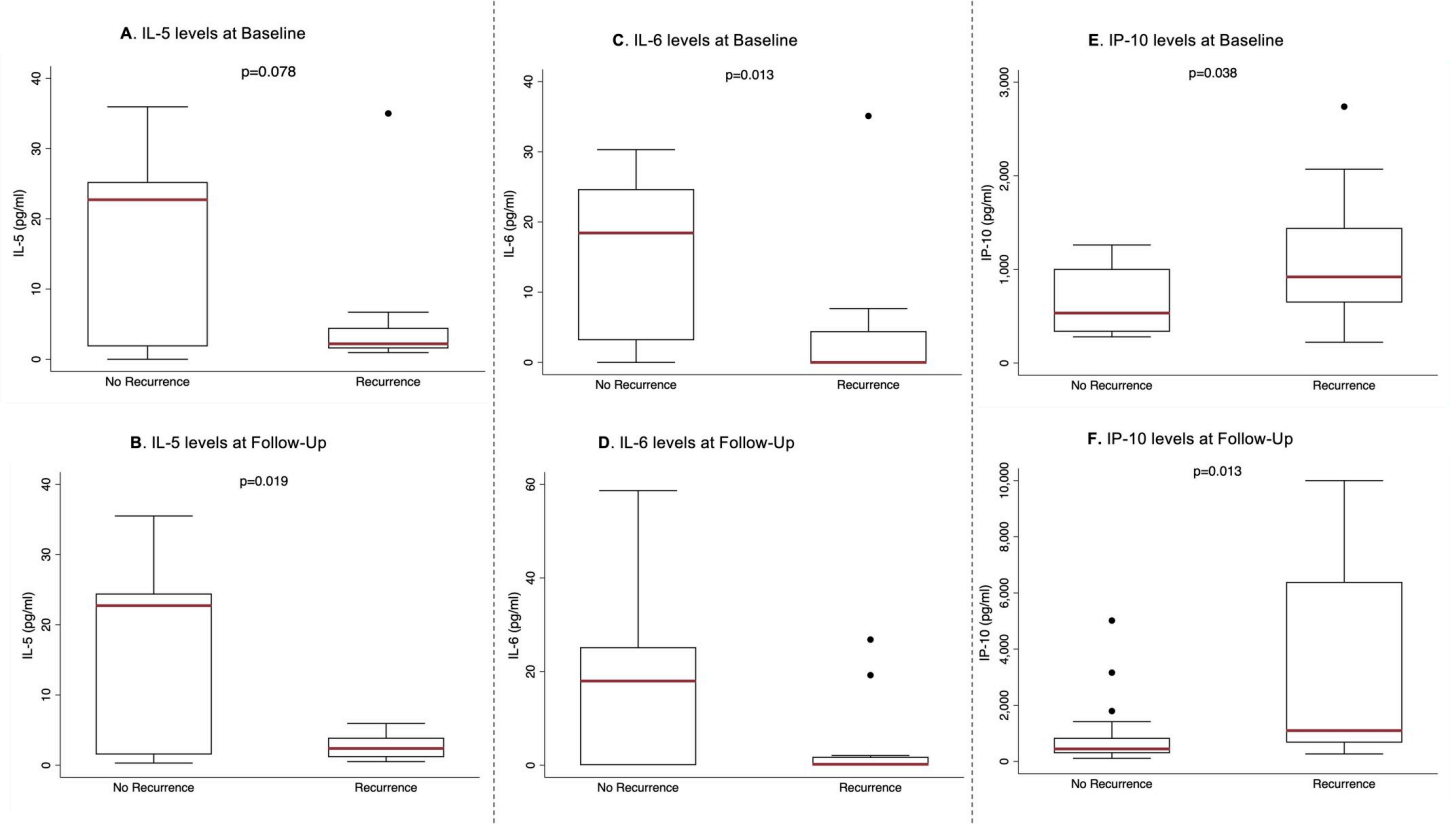
Baseline and follow-up cytokines and chemokines in individuals with no recurrence (sustained remission) compared to those with KS recurrence. A) No difference in baseline IL-5; B) IL-5 significantly higher at the time of follow-up in individuals with sustained remission compared to those with KS recurrence; C) IL-6 significantly higher at baseline in individuals with sustained remission compared to those with KS remission; D) IL-6 significantly higher at the time of follow-up in individuals with sustained remission compared to those with KS recurrence; E) IP-10 significantly lower at baseline in individuals with sustained remission compared to those with KS remission; F) IP-10 significantly lower at the time of follow-up in individuals with sustained remission compared to those with KS recurrence.

We quantified and compared plasma levels of cytokines and chemokines including IL-4, IL-5, IL-6, IP-10, VEGF-A, and IFNγ at baseline, at the time of follow up and between baseline and follow-up between individuals that had KS recurrence versus those who had sustained remission. These are cytokines and chemokines that have been previously associated with humoral immune response, cell growth and survival, tumor proliferation and survival, chemotaxis, and up- or down-regulation of cancer-related inflammation [11–13]. Interleukin-5 (IL-5) was significantly higher at the time of follow-up in individuals that had sustained remission compared to those who had KS recurrence. Interleukin-6 (IL-6) was significantly higher at baseline and at the time of follow-up in individuals who had sustained remission compared to those who had disease recurrence. The chemokine IP-10 was significantly lower at baseline and at the time of follow-up in individuals that had sustained remission compared to those who had disease recurrence (Fig 1). The cytokines IFNγ, IL-4, and VEGF were not significantly different between individuals who had sustained remission versus those who had disease recurrence (Table 3).

**Table 3:** Comparison of cytokine/chemokine levels and HIV-related parameters in individuals experiencing recurrence versus those in remission.

	No Recurrence			Recurrence				
	Baseline	At Follow-up	P value^#^	Baseline	At Follow-up	P value^#^	P value*	P value**
**IFN-γ**	33.1 [0–53.4]	30.7 [0–50]	0.81	4.7 [0–11.1]	6.4 [0.7–11.1]	0.78	0.08	0.10
**IL-4**	0 [0–56.2]	0 [0–83.1]	0.60	0 [0–0]	3.4 [0–42.9]	0.14	0.11	0.81
**IL-5**	22.7 [1.8–25.2]	22.7 [1.5–24.4]	0.78	2.2 [1.5–4.5]	2.4 [1.1–3.9]	0.24	0.08	**0.02**
**IL-6**	18.4 [3.2–24.7]	18.0 [0–25.2]	0.96	0 [0–4.5]	0.18 [0–1.8]	0.75	**0.01**	**0.03**
**IP-10**	534 [334–1006]	446 [293–841]	0.40	920 [646–1444]	1098 [669–6393]	0.53	**0.04**	**0.01**
**VEGF**	0 [0–66.5]	0 [0–81.6]	0.43	0 [0–85.6]	31.3 [0–67.2]	0.94	0.83	0.68
**CD4 Count**	231 [140–314]	282 [187–384]	0.09	233 [162–293]	285 [145–349]	0.48	0.97	0.37
**HIV Viral Load**	0 [0–0]	0 [0–30]	0.86	113 [0–252241]	152 [0–721]	0.09	**0.004**	**0.025**

All cytokine/chemokine values in pg/ml, CD4 counts in cells/μl, and HIV viral load in copies/ml.

* = comparison between baseline values

** = comparison between follow up values

# = intragroup comparison between baseline and follow-up values. [Interquartile range]. P values in bold are statistically significant.

### Comparison of CD4 counts and HIV viral loads

There was no significant difference in CD4 counts at baseline and at the time of follow-up between individuals who had sustained KS remission and those who had KS recurrence. The HIV viral load was significantly higher at baseline and at the time of follow-up in individuals who had KS recurrence compared to those who had sustained KS remission (Table 3). There was no correlation between baseline CD4 counts and HIV viral load in the entire cohort (⍴ = 0.002; p = 0.99), in the group experiencing KS recurrence (⍴ = 0.06; p = 0.71), and in the group with sustained KS remission (⍴ = -0.06; p = 0.86). There was no correlation between follow-up CD4 counts and HIV viral loads in the entire cohort (⍴ = -0.23; p = 0.16), in the group experiencing KS recurrence (⍴ = 0.02; p = 0.95), and in the group with sustained KS remission (⍴ = -0.26; p = 0.21).

## Discussion

This study investigated the factors that are associated with recurrence of HIV-associated KS. Sociodemographic factors including age, sex, smoking status, and alcohol consumption were not associated with KS recurrence. Also, clinical factors including duration of HIV infection, duration of ART uptake, history of multiple KS lesions, history of mucous membrane involvement, and persistent lymphedema were not associated with KS recurrence. These findings suggest that clinical and sociodemographic factors are possibly poor predictors of KS recurrence. However, a larger sample size with additional clinical characteristics, including patients with a history of visceral KS may be required to conclude on these findings.

The cytokine IL-5 was significantly higher at the time of follow-up in individuals who had sustained remission compared to those who had KS recurrence. IL-5 is a Th2 cytokine that promotes terminal differentiation of B cells into antibody-secreting plasma cells, and is also an important factor in the differentiation and maturation of eosinophils [14, 15]. In a previous study, we observed that higher levels of plasma IL-5 were associated with a good treatment outcome for ART-treated HIV-associated KS [12]. However, in that study, IL-5 was not correlated with KSHV neutralizing antibodies (nAb). We did not investigate KSHV nAb in this study, however, this may be an important area of future research to determine whether or not IL-5 promotes KSHV nAb production that may potentially protect against KS recurrence. Nevertheless, high plasma IL-5 is a marker of sustained KS remission.

Interleukin-6 was significantly higher at baseline and at the time of follow-up in individuals with sustained remission compared to those who had KS recurrence. IL-6 is a pleiotropic cytokine with both pro-inflammatory and anti-inflammatory effects [16]. In cancer, IL-6 appears to have a dual role of either driving tumor growth or promoting anti-tumor immunity [17]. As a tumor driver, IL-6 promotes tumor cell proliferation, survival, angiogenesis, and evasion of immune surveillance [17, 18]. Anti-tumor effects of IL-6 include lymphocyte trafficking to lymph nodes, proliferation, activation, and polarization to phenotypes that have anti-tumor effects [19]. In KS, IL-6 has been found to be elevated in both HIV-associated and non-HIV-associated KS [20]. In addition, IL-6 was observed to be higher among individuals with early-stage HIV-associated KS who had disease progression compared those who responded favorably to antiretroviral therapy [12]. Our findings, in comparison to previous studies highlight the pleotropic effects of IL-6. It is possible that in the initial presentation of HIV-associated KS, and for ART-treated KS, IL-6 is a driver of KS disease development and progression. On the other hand, persistently high IL-6 levels after initial control of HIV-associated KS with cancer chemotherapy seems to be important for maintenance of disease remission, possibly through promoting anti-tumor effects. Furthermore, KSHV is known to produce the viral homolog of IL-6 (vIL-6) which promotes KSHV lytic replication and KS tumor proliferation [21]. It is important for future studies to explore the role of vIL-6 in KS re-development.

The chemokine IP-10, also known as CXC motif chemokine ligand 10 (CXCL10), was significantly higher at baseline and at the time of follow-up in individuals who had KS recurrence compared to those who had sustained remission. IP-10 is a chemokine that intensifies inflammation by recruiting leukocytes to tissues. IP-10 is associated with HIV infection and disease progression, including the suppression of T cell responses in HIV-infected individuals who are on ART [22, 23]. In cancer, IP-10 can either inhibit or promote tumors depending on the corresponding spliced variant of the its receptor CXCR3 [24]. CXCR3-A promotes tumor cell proliferation while CXCR3-B inhibits tumor proliferation [24]. IP-10 has been observed to inhibit the growth of malignancies such as cervical cancer through anti-angiogenic and anti-viral mechanisms [25], while it has been observed to promote growth of malignancies such as breast cancer through the CXCR3-A receptor variant [24]. For KS, IP-10 seems to have a tumor promoting effect. We have previously observed persistently high IP-10 levels in ART-treated individuals with HIV-associated KS who have disease progression, while those who have tumor regression have a significant decrease in IP-10 levels [12]. In other previous studies, we have observed an upregulation of IP-10 gene expression in KS lesions compared to non-affected tissues from the same individuals [26]. Individuals on ART and treated for HIV-associated KS who have persistently high IP-10 levels need more close monitoring for KS recurrence. In addition, these individuals may be good candidates for maintenance chemotherapy upon responding to the recommended chemotherapy regimens. Furthermore, anti-IP-10 treatment may be a potentially effective future treatment for HIV-associated KS, especially for individuals at risk of recurrence and those who respond poorly to first-line chemotherapy.

A decrease in CD4 counts to significantly low levels below 200 cells/μl is a sign of progression to AIDS in HIV-infected individuals [27]. Low CD4 counts predispose HIV-infected individuals to opportunistic infections such as tuberculosis, and HIV-associated malignancies such as KS [28]. Antiretroviral therapy is key in reversing the HIV-induced immunosuppression, with an associated increase in CD4 counts that prevents and even treats opportunistic infections [29, 30]. In a previous study, we observed that when HIV-infected individuals with KS were commenced on ART, some had disease progression while others had disease regression despite all of them having virological control of the HIV [6]. In that study, there was no significant difference in CD4 counts at baseline and during follow-up in individuals who had disease progression compared to those who had tumor regression [6]. In this current study, we have observed that there was no significant difference in CD4 counts at baseline and time of follow-up in individuals that had sustained KS remission compared to those who had KS recurrence. Also, there was no significant change in CD4 counts over time within individuals that had KS recurrence and in those who had sustained remission.

The HIV viral load was significantly higher at baseline and at the time of follow-up in individuals who had KS recurrence compared to those who had sustained remission. Actually, the median HIV viral load was undetectable at both baseline and follow-up in individuals who had sustained remission while it was detectable and slightly increased from baseline levels in those who had KS recurrence. Viral load is a good measure of response to ART, and helps in the monitoring of ART adherence and efficacy [31]. High HIV viral loads are seen in individuals who are ART naïve and in those who have ART treatment failure [31]. HIV viral load can be defined as suppressed (below a certain threshold) or undetectable (cannot be detected with standard available tests). Definitions for suppressed HIV viral loads vary, with the CDC being <200copies/ml while the WHO definition is <1000copies/ml [32, 33]. In our study, individuals who had suppressed (both CDC and WHO definition) but persistently detectable HIV viral loads experienced KS recurrence, while those who had suppressed and undetectable viral loads had sustained KS remission. This supports the idea of interpreting any sustained, detectable viremia (viral load >50copies/ml) as treatment failure necessitating a switch of the regimen as is the case in some resource-rich settings, because resistance mutations may emerge even with low-level replication [34]. Persistently detectable HIV viral loads due to low-level replication can give rise to mutations resulting in virological failure and chronic immune activation which may increase the risk of cancer and cardiovascular diseases [35–37]. Clinicians should be cautious of individuals presenting with suppressed but detectable HIV viral loads and, if possible, conduct HIV resistance testing to determine whether there is ART resistance. However, a follow-up study with a larger sample size is warranted to confirm whether or not HIV viral load is a good marker of KS disease recurrence necessitating ART resistance-testing or change of the ART regimen.

### Study limitations

The small sample size is a limitation when determining the clinical and sociodemographic predictors of KS recurrence. A larger cohort is required to confirm our observations in this regard. In addition, individuals with a history of visceral KS were not included as part of this cohort, as visceral KS may be associated with recurrence. Furthermore, some baseline data from before chemotherapy is also lacking as we only recruited study participants after they went into complete remission. It would be interesting for future studies to compare those in partial remission to those who achieve complete remission. Also, we were unable to determine the trends in the biomarkers over time.

## Conclusions

For individuals with HIV-associated KS achieving complete remission after chemotherapy, sociodemographic and clinical features are not helpful in predicting disease recurrence. The findings from this study suggest that high IL-5 and IL-6 are associated with sustained remission, while high IP-10 is associated with recurrence of HIV-associated KS. Also, the findings suggest an association between persistently detectable but suppressed HIV viral loads with KS recurrence. A validation of these findings in larger cohorts is needed.

## Supporting information

S1 File. Dataset Individual data on all collected information including sociodemographic, clinical, HIV viral loads, CD4 counts, and cytokine measurements. (XLSX)
